# Supplementary material for: Causal Relationship between Aging and Anorexia Nervosa: A White-Matter-Microstructure-Mediated Mendelian Randomization Analysis
Source: Biomedicines. 2024 Aug 16;12(8):1874. doi: 10.3390/biomedicines12081874 (PMC11351342; doi:10.3390/biomedicines12081874)
Supplement: Supplementary file 1 [file biomedicines-12-01874-s001.zip › Supplementary Figure/Figure S9/Leaveoneout plot of MR analysis for ubm-b-1701 to AN.pdf]

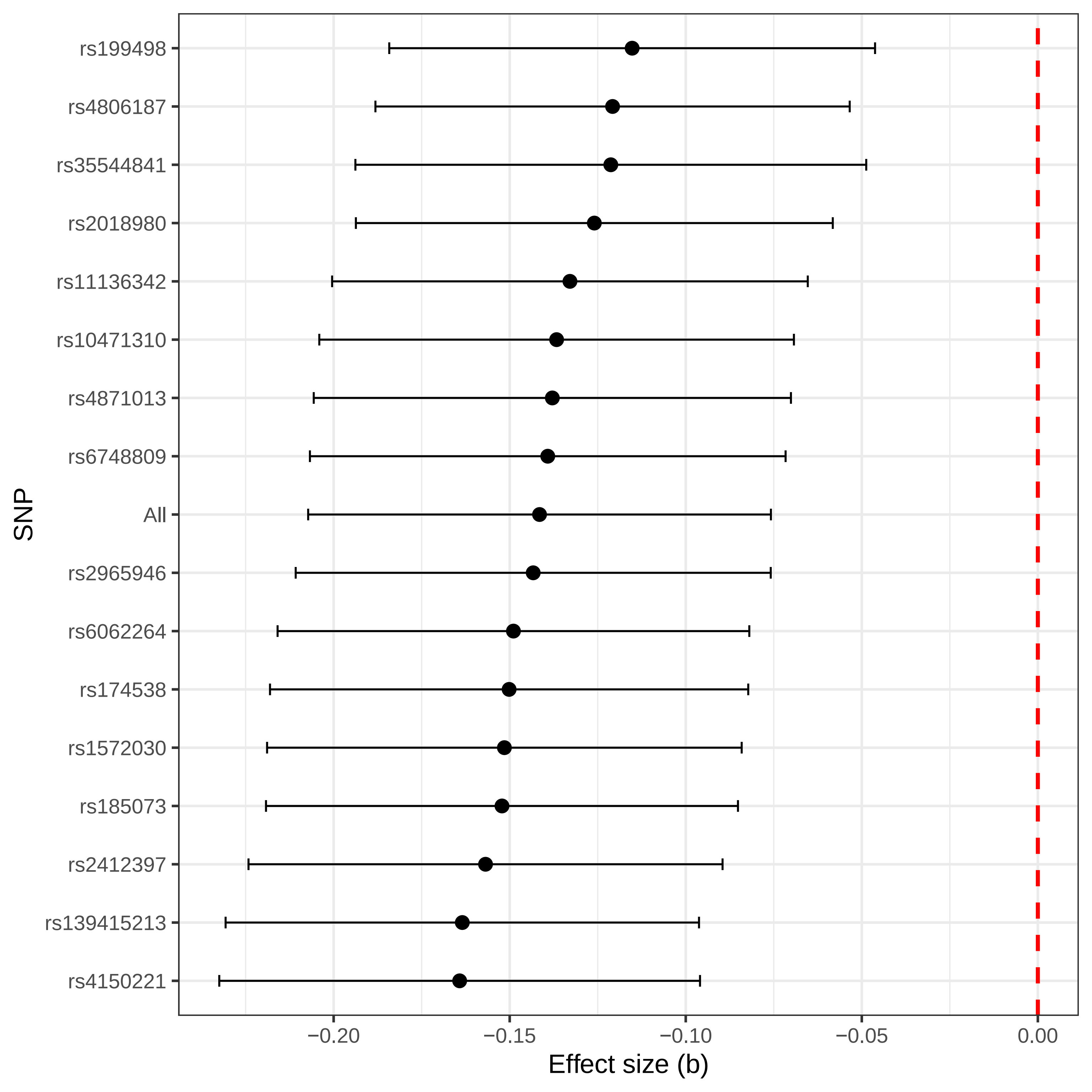

rs199498
rs4806187
rs35544841
rs2018980
rs11136342
rs10471310
rs4871013
rs6748809
All
rs2965946
rs6062264
rs174538
rs1572030
rs185073
rs2412397
rs139415213
rs4150221
SNP
−0.20
−0.15
−0.10
−0.05
0.00
Effect size (b)
